# Application of Metal–Organic Framework-Based Electrochemiluminescence Sensors for Mycotoxin Detection in Food

**DOI:** 10.3390/bios16060329

**Published:** 2026-06-09

**Authors:** Tong Zhang, Xinyu Chen, Qiangqiang Wang, Shuyue Xing, Dan Wu

**Affiliations:** 1 Institute of Traditional Chinese Medicine Preparations, Shandong Academy of Chinese Medicine, Jinan 250014, China; zhangtong@sacm.ac.cn (T.Z.);; 2 Key Laboratory of Interfacial Reaction & Sensing Analysis in Universities of Shandong, School of Chemistry and Chemical Engineering, University of Jinan, Jinan 250022, China

**Keywords:** metal–organic frameworks, electrochemiluminescence sensors, mycotoxins

## Abstract

Mycotoxins are toxic secondary metabolites produced by filamentous fungi, which extensively contaminate agricultural products such as grains and nuts. Common mycotoxins, including aflatoxin B1, ochratoxin A, and deoxynivalenol, can induce liver cancer, kidney damage, neural tube defects, and immune suppression, necessitating highly sensitive detection methods to ensure food safety. Conventional techniques are limited by complex procedures and insufficient sensitivity. Electrochemiluminescence (ECL), owing to its high sensitivity, low background signal, and rapid response, has emerged as a promising strategy for mycotoxin analysis. In this context, metal–organic frameworks (MOFs), with their high surface area and tunable structures, have been widely employed in ECL sensors to improve sensing performance. This review summarizes the construction strategies of MOF-based ECL sensors, the diverse functional roles of MOFs in ECL sensing, the associated sensing mechanisms, and the applications of these sensors for the detection of mycotoxins in food. Current challenges, including material stability, sensor reproducibility, and practical applicability, are discussed, and future directions are outlined. Particular emphasis is placed on the development of stable MOF materials, their integration into portable and intelligent ECL sensing platforms, and the establishment of standardized and scalable production methods to enable practical food safety monitoring.

## 1. Introduction

Mycotoxins are toxic secondary metabolites produced by filamentous fungi that extensively contaminate various food products. These contaminants pose serious threats to human health through genotoxicity, mutagenicity, and hepatotoxicity [1,2,3]. Aflatoxin B1 (AFB1), ochratoxin A (OTA), zearalenone (ZEN), deoxynivalenol (DON), and fumonisin B1 (FB1) are the most frequently detected mycotoxins, with occurrences reported in grains, medicinal and edible plants, and fermented beverages such as beer [4,5,6]. AFB1 is the most prevalent and toxic among aflatoxins and has been classified as a Group 1 human carcinogen by the International Agency for Research on Cancer. Even at trace levels (here referring to very low concentrations relevant to food contamination and safety monitoring) [7,8], AFB1 exposure has been associated with carcinogenicity, organ toxicity, and neurotoxicity. AFB1 can induce cancer, organ toxicity, and neurotoxicity [9,10,11,12,13]. OTA exhibits potent nephrotoxicity and developmental toxicity [14,15], while ZEN and its metabolites, widely present in corn and wheat, possess estrogenic activity that can disrupt reproductive and endocrine functions [16,17]. Exposure to DON affects host metabolism, immune function, and intestinal barrier integrity, leading to metabolic disorders, immune suppression, and oxidative stress [18,19,20]. Given these health risks, systematic monitoring of mycotoxins is of critical importance for strengthening food safety defenses and safeguarding public health.

Liquid chromatography-tandem mass spectrometry (LC-MS/MS) and enzyme-linked immunosorbent assay (ELISA), among other traditional methods, have been widely used for mycotoxin detection [21,22,23]. LC-MS/MS requires laborious sample pretreatment and consumes large amounts of chemical reagents, which may pose environmental concerns. Additionally, this method demands high-performance instrumentation and specialized operator expertise, making it difficult to implement in resource-limited laboratories [24]. While ELISA is widely used for mycotoxin screening because of its operational simplicity, high throughput, and good sensitivity, its practical application may still be limited by susceptibility to external environmental factors, poor enzyme stability, potential cross-reactivity, matrix dependence, and the fact that most commercial kits are designed for single-mycotoxin, single-use analysis, which may increase the cost of multi-mycotoxin detection [25,26]. Numerically, the limits of detection (LODs) obtained by conventional LC-MS/MS are generally at the ng/mL to μg/kg level, while those of ELISA are commonly at the ng/mL to μg/kg level or higher [22]. In sharp contrast, metal–organic framework-based electrochemiluminescence (MOF-ECL) biosensors can achieve LODs at fg/mL to pg/mL levels, which are comparable to or even lower than those of LC-MS/MS and ELISA under the same detection conditions [27]. These factors collectively restrict the widespread application of LC-MS/MS and ELISA in routine monitoring systems for large-scale food production. Therefore, there is a pressing need to develop alternative methods that combine rapid response, high sensitivity, and on-site applicability for mycotoxin surveillance.

**Figure 1:**
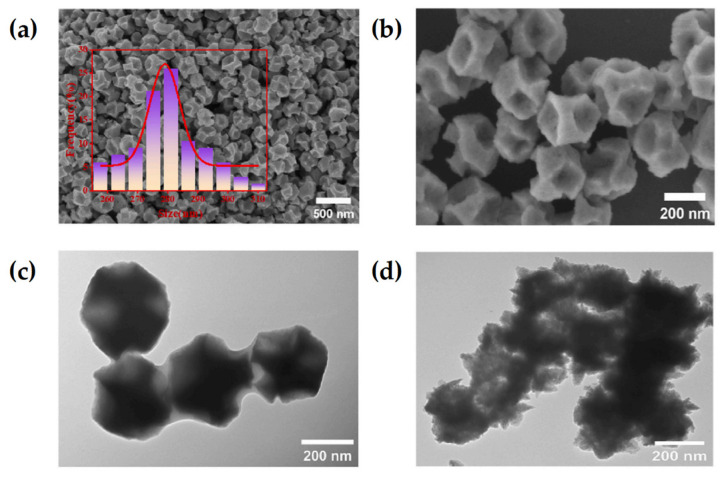
Representative SEM/TEM images of MOF-based sensing materials used in ECL biosensors: (**a**,**b**) SEM images of Pd-Cu_2_O; (**c**) TEM image of Pd-Cu_2_O; (**d**) TEM image of PtNPs/Pd-Cu_2_O. Reproduced with permission from Ref. [41]. Copyright 2025, Elsevier.

ECL sensors have been widely recognized for their excellent sensitivity, low background interference, and suitability for portable devices, supporting selective, ultrasensitive, and high-throughput detection of target analytes [28,29]. The low-background characteristic of ECL mainly originates from the absence of an external excitation light source, which minimizes optical interference such as autofluorescence and scattered light. For signal-off ECL sensors, this feature is particularly beneficial for maintaining a high signal-to-noise ratio and enabling reliable discrimination of emission attenuation from background noise, although it does not directly amplify the analytical signal. Nevertheless, traditional ECL sensing platforms still present notable limitations. Representative ECL luminophores include traditional organic luminophores such as luminol and its derivatives, and classical metal-complex emitters such as tris(2,2′-bipyridine)ruthenium(II) (Ru(bpy)_3_^2+^). Luminol is a classic organic molecule widely used as an ECL luminophore in aqueous media because of its low oxidation potential, low cost, nontoxic nature, and high light-emitting quantum yield. However, the commonly used luminol/H_2_O_2_ ECL system may be restricted by the short-time radical stability, low quantum efficiency, and instability of H_2_O_2_ under certain conditions [30]. In addition, Ru(bpy)_3_^2+^ shows high ECL efficiency and chemical stability, but can suffer from aggregation-caused quenching upon aggregation or crystallization, which weakens its ECL efficiency in solid-state applications [31]. These issues motivate the development of more robust ECL platforms based on functional materials such as MOFs. In the field of mycotoxin detection, another important class of highly sensitive biosensors is based on aptamer- or antibody-functionalized metal nanoparticles, in which the toxin signal is generated through the surface-enhanced Raman scattering (SERS) effect [32]. According to the reviewed literature, SERS biosensors based on aptamer or antibody-functionalized metal nanoparticles are also capable of rapid and sensitive toxin analysis; however, their performance is more susceptible to metal nanoparticle aggregation, limited colloidal stability, and the difficulty of controlling the chemical or physical fixation of analytes on metal surfaces [33]. Such limitations may compromise signal reproducibility and practical applicability, especially in complex food matrices. Metal–organic frameworks (MOFs) are a class of crystalline porous materials formed by the coordination bonds between metal ions or metal clusters and organic bridging ligands, exhibiting characteristics such as high specific surface area, tunable pore size, and structural designability, according to the International Union of Pure and Applied Chemistry classification, the pore structures of MOF materials are generally categorized as micropores (<2 nm) and mesopores (2–50 nm). In ECL biosensing applications, such tunable porosity is beneficial for facilitating analyte diffusion, promoting target enrichment, and improving the loading efficiency of luminophores and biorecognition elements [34]. By rationally selecting metal centers and organic ligands, the pore size and overall topological structure of the materials can be precisely controlled [35]. With the development of novel functional materials such as MOFs, covalent-organic frameworks (COFs), metal oxide nanomaterials, and carbon-based nanomaterials, the applicability of this detection technology has been significantly enhanced [36,37]. These structural features make MOFs particularly attractive for overcoming interfacial limitations in trace biosensing, because they can promote target enrichment, improve the immobilization of recognition elements and luminophores, and facilitate electron transfer. Compared with metal nanoparticle-based SERS biosensors, MOF-ECL platforms combine the intrinsically low-background and low-interference nature of ECL with the structural advantages of MOFs, and may therefore provide a more robust analytical framework and sensitivity that is highly competitive for ultratrace mycotoxin determination in complex food systems [38,39,40]. As a complementary visualization of these structural advantages, representative SEM/TEM images of MOF-based sensing materials are presented in Figure 1 to provide intuitive insight into their morphology and nanoscale architectures.

In mycotoxin detection, the well-defined pore structure facilitates the rapid diffusion and enrichment of target toxin molecules. It also improves the immobilization efficiency of recognition elements, thereby enhancing sensor sensitivity and detection reproducibility [42,43]. In addition to serving as a luminescent emitter support, MOFs also function as intrinsic ECL emitters and signal amplification platforms. For instance, through diverse signal amplification strategies such as co-reactant acceleration and nanozyme catalysis, MOFs have significantly enhanced the detection sensitivity and anti-interference capability of sensors toward toxins such as AFB1 and OTA, gradually promoting the development of multi-toxin simultaneous detection technologies [41,44].

**Figure 2:**
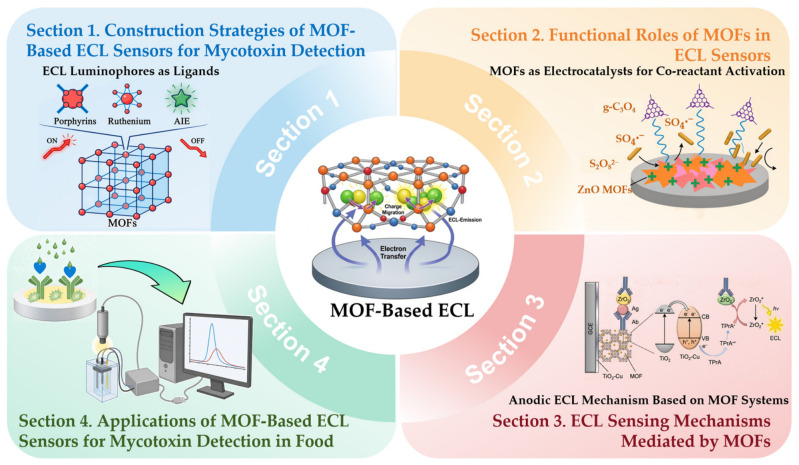
Design strategies, mechanisms, and mycotoxin detection applications of MOF-based ECL sensors. The symbol * represents the excited state.

Despite the significant advantages of MOF-ECL sensors, their practical applications are still limited by several bottlenecks, including aqueous stability, signal reproducibility, uniformity in batch production, and interference from complex food matrices [45,46]. Recent reviews have provided broad overviews of ECL sensors for mycotoxin monitoring in food [47]. In contrast, the present review focuses on MOF-based ECL sensing systems and highlights the roles of MOFs in sensor construction and performance enhancement (Figure 2). Current challenges and future directions are also discussed, particularly with regard to material stability, sensor reproducibility, practical applicability, and the integration of MOF-based ECL sensors into portable and intelligent platforms.

## 2. Construction Strategies of MOF-Based ECL Sensors for Mycotoxin Detection

Mycotoxin detection demands high sensitivity, making ECL-active MOFs ideal sensing platforms owing to their structural designability, tunable component coupling, porosity, and electrocatalytic characteristics [48]. The use of such MOFs in sensor construction not only significantly enhances the immobilization efficiency of recognition elements, such as antibodies and aptamers [49,50], but also eliminates the need for additional coreactant promoters, thereby simplifying the operational procedure and improving sensor stability [51]. Currently, synthetic strategies for tailoring ECL-active MOFs for detection applications primarily encompass three approaches: employing ECL luminophores as ligands, in situ encapsulation of guest molecules, and post-synthetic modification.

### 2.1. ECL Luminophores as Ligands

MOFs possess well-defined molecular configurations and tunable pore structures. The incorporation of intrinsic luminescent ligands, such as porphyrins, ruthenium-based luminophores, and aggregation-induced emission (AIE) luminophores, into the MOF synthesis system provides an ideal pathway for the preparation of functionalized MOF materials. The embedding of these luminescent ligands not only endows MOFs with enhanced physicochemical properties but also significantly broadens their application potential in ECL sensor systems, laying a solid material foundation for the development of highly sensitive mycotoxin ECL sensors.

**Figure 3:**
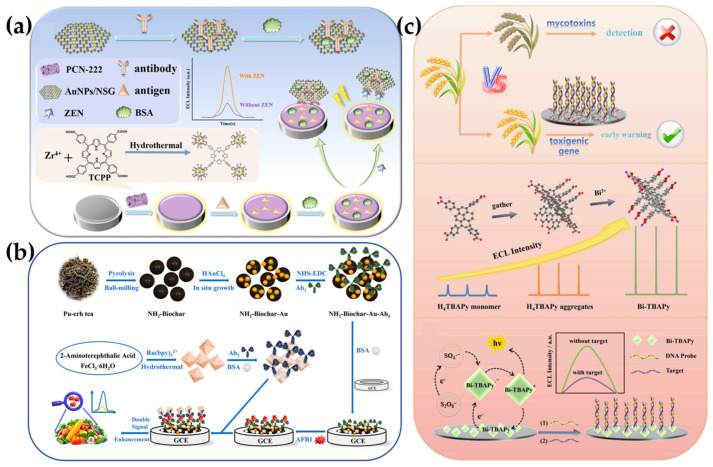
Schematic illustration of different construction strategies for MOF-based ECL sensors for mycotoxin detection: (**a**) ECL luminophores as ligands. Reproduced with permission from Ref. [54]. Copyright 2023, Royal Society of Chemistry. (**b**) In situ encapsulation of guest molecules. Reproduced with permission from Ref. [68]. Copyright 2023, MDPI. (**c**) post-synthetic modification. Reproduced with permission from Ref. [82]. Copyright 2025, Elsevier. The symbol * represents the excited state.

Porphyrin compounds are large macromolecular heterocyclic structures composed of four pyrrole subunits interconnected via methine bridges (=CH–) at their α-carbon atoms [52]. In recent years, research has explored the incorporation of mycotoxins or their structural analogs into porphyrin-based MOF systems to develop highly sensitive biosensing platforms for toxin detection or environmental monitoring. For instance, tetrakis(4-carboxyphenyl)porphyrin (TCPP) has been integrated as an organic ligand into various MOFs, where the MOF structure not only prevents porphyrin aggregation-induced quenching but also leverages the porphyrin’s large π-conjugated system to endow the material with efficient ECL performance. In this representative system, the TCPP-containing MOF acts as the ECL-emitting framework and is combined with an immunorecognition interface for competitive ECL-resonance energy transfer (RET) detection of ZEN, highlighting the advantage of incorporating luminophores directly as MOF ligands (Figure 3a). This enables trace-level, highly sensitive detection of mycotoxins such as OTA and ZEN through either direct competitive immunoassays or ECL-RET mechanisms combined with competitive immunoassays [53,54]. Alternatively, the catalytic activity of TCPP can be harnessed to impart excellent peroxidase-like activity to the material. The MOF structure further suppresses iron-porphyrin self-dimerization, enhances material dispersibility, and improves the accessibility of active sites, ultimately enabling ultrasensitive detection of mycotoxins [55].

Functionalized MOF materials based on ruthenium-based luminophores have opened new pathways for the highly sensitive trace detection of mycotoxins, owing to their excellent ECL performance and target recognition capabilities. Kent et al. [56] first reported ruthenium-based MOFs. In recent studies, ruthenium-based ligands have been coordinated with indium(III) to construct RI-MOFs, where charge transfer from In^3+^ to the ruthenium center enhances the efficiency of co-reactant-free ECL annihilation. These materials, used as luminophores in combination with DNA walker-based signal amplification, enable highly sensitive detection of AFB1 [57]. Ru-MOFs can also serve as ECL-RET systems with Bi_2_S_3_ nanorods, resulting in ECL signal quenching. The specific binding of DON to its aptamer can relieve this quenching and restore the ECL signal, enabling the construction of an “off-on” ECL aptasensor for the highly sensitive detection of DON in wheat and corn [58].

Traditional organic luminophores often suffer from aggregation-caused quenching (ACQ), where their luminescence is significantly weakened or even completely quenched in the aggregated state. In contrast, AIE materials exhibit the inverse behavior, characterized by “emission in the aggregated state and quenching in the dispersed state,” which can be explained by the restriction of intramolecular motions (RIM) mechanism. Anchoring AIE chromophores within MOFs not only provides a platform for a deeper understanding of the AIE mechanism but also creates opportunities to explore highly attractive optical properties in novel matrices. When AIE molecules coordinate with metal centers, their internal vibrations or rotations are restricted, leading to brighter fluorescence with enhanced intensity and quantum yield [59]. In AIE materials, the phenyl rotors are fully replaced by coordinating groups within the MOF, forming a rigid structure that enables a high level of RIM. Moreover, the confined pore space within the framework effectively restricts intramolecular motion of AIE molecules, efficiently eliminating ACQ caused by aggregation [60]. In the field of mycotoxin detection, the performance advantages of AIE-MOF materials have been well validated. For instance, Zr-TCPB synthesized using the AIE molecule 1,2,4,5-tetrakis(4-carboxyphenyl)benzene (H_4_TCPB) as a ligand enhances aggregation-induced electrochemiluminescence (AIECL) performance by restricting the intramolecular motion of H_4_TCPB within its framework. Combined with a dual signal amplification strategy, this material enables ultrasensitive detection of AFB1 [61]. Furthermore, leveraging the AIE property to restrict ligand intramolecular motion and reduce non-radiative decay, while combining it with an antenna effect for efficient energy absorption and transfer to Eu^3+^, significantly improves the ECL stability and intensity of Eu-MOFs, making them high-performance AIECL emitters. Modifying the electrode with Au@Sn_3_O_4_ nanoflowers as a co-reactant accelerator not only enhances electron transfer and catalytic efficiency, amplifying the ECL signal, but also provides a high-surface-area substrate for antigen immobilization. Based on this, an AIECL immunosensor was constructed, enabling ultrasensitive detection of DON in wheat and corn through a competitive immunoassay [62].

### 2.2. In Situ Encapsulation of Guest Molecules

During MOF construction, the in situ synthesis strategy is used to encapsulate luminescent species within the MOF matrix, delivering distinct benefits: enhanced luminophore loading, improved luminescence stability, optimized charge/mass transport, and synergistic enhancement [63,64,65,66]. More than a simple loading method, this strategy provides a confined microenvironment to regulate the dispersion, stability, and interfacial behavior of molecular emitters, which is especially important for metal-complex luminophores suffering from poor aqueous compatibility or unstable immobilization.

For instance, the directional encapsulation of an iridium(III)-polyimine complex within an in situ grown Hf-MOF nanosheet film effectively converted a water-incompatible molecular emitter into a stable aqueous-phase ECL interface. The enhanced red ECL emission arose from the uniform confinement of Ir_2_PD, increased loading, suppressed aggregation, and improved electrode adhesion [67]. The incorporation of Ru(bpy)_3_^2+^ into MIL-88B(Fe)-NH_2_ reflects another dimension of in situ encapsulation, namely the coupling of luminophore enrichment with framework-mediated reaction promotion. In this system, MIL-88B(Fe)-NH_2_ provides abundant pores for guest loading and mass transport, while its amino groups and Fe-based nodes facilitate the activation of the co-reactant K_2_S_2_O_8_. Thus, the improved ECL response does not arise solely from a higher local concentration of Ru(bpy)_3_^2+^, but from the coordinated contributions of emitter confinement, co-reactant diffusion, and catalytic activation. In the representative immunosensor shown in Figure 3b, the biochar-Au-Ab_1_ interface is used for AFB1 capture, whereas MIL-88B(Fe)-NH_2_@Ru(bpy)_3_^2+^-Ab_2_-BSA serves as the ECL signal probe. When used as a signal probe in an AFB1 immunosensor, this composite illustrates how MOF encapsulation can convert a conventional Ru-based ECL system into a framework-assisted signal amplification platform [68]. These examples indicate that in situ encapsulation should be viewed as a microenvironment-engineering strategy, integrating emitter stabilization, signal amplification, and sensing-function construction within one MOF-based platform.

### 2.3. Post-Synthetic Modification

Pre-synthetic strategies often suffer from limited flexibility in functional group incorporation and may not adequately address intrinsic material drawbacks, such as monotonous porosity and poor processability. To meet the specific functional requirements of different applications and broaden the structural diversity and application scope of materials, post-synthetic incorporation of guest species is widely employed to construct ECL-active MOFs [69,70,71,72]. This approach offers strong functional versatility, enables precise structural control while preserving the high surface area and porous nature of MOFs, is applicable to various MOF systems, and significantly enhances material stability and practical value. It also provides flexible and controllable synthesis with reduced preparation costs. Through covalent or coordination-based modification, diverse functionalities such as MOF-polymer composites, hierarchical pore construction, and biofunctionalization can be achieved [73].

Covalent modification of MOFs refers to a strategy in which functional molecules are covalently bonded to MOFs under mild reaction conditions, targeting well-defined active sites on organic linkers or surfaces, such as amino or hydroxyl groups [74,75]. This is typically achieved through surface covalent grafting or photo-induced covalent crosslinking, directly altering the functional groups of ligands or the chemical properties of the MOFs. The core advantages of this approach include well-defined modification sites, high bonding stability, and the ability to precisely tune the pore environment of MOFs [76,77,78]. For instance, studies have addressed the limitations of pure polydopamine (PDA) quenchers, such as strong electronegativity, low specific surface area, and limited radical scavenging capacity, by covalently integrating PDA with ZIFs. This covalent composite enhances quenching performance and ECL sensing efficiency. Importantly, the covalent modification preserves the porous structure and surface active sites of ZIFs, facilitating subsequent non-covalent coupling with anti-OTA monoclonal antibodies (mAbs) to form immunoprobes [79].

Coordination modification of MOFs involves utilizing the coordination activity of metal nodes (e.g., Zr^4+^, Zn^2+^) to anchor molecules containing coordinating groups (e.g., carboxyl, phosphate) onto Secondary Building Units. The key advantages of this strategy include the preservation of the internal porous structure and the compatibility with surface functionalization requirements [80,81]. Min et al. [82] assembled Bi^3+^ with the organic ligand H_4_TBAPy through coordination bonds to form the Bi-TBAPy MOFs. This composite suppressed the ACQ effect commonly observed in traditional organic emitters through a rigid coordination network, while also offering low toxicity and low cost. After the formation of Bi-TBAPy, DNA probes were further introduced onto the sensing interface to recognize the OTA-producing halogenase gene fragment, linking the post-synthetic biofunctionalization of the MOF emitter with gene-level ECL detection (Figure 3c). The detection target was shifted from “pre-formed OTA toxin” to “key gene involved in OTA biosynthesis (halA gene fragment)”, enabling a transition from “retrospective detection” to “proactive early warning”. This approach provides a novel strategy for the source control of mycotoxin contamination.

## 3. Functional Roles of MOFs in ECL Sensors

The functional role of MOFs in ECL sensing systems directly determines their material design strategies and synthetic approaches. Based on their interaction modes with ECL emitters, co-reactants, and energy-transfer processes, MOFs can be classified into five functional categories: ECL emitter carriers, ECL emitters, co-reactant promoters, ECL-RET energy-transfer pairs, and multifunctional integrated materials.

### 3.1. MOFs as Carriers

The pore topology of MOFs endows them with dual core functionalities. First, they can accelerate the mass transfer kinetics of co-reactants, and second, they ensure the full exposure and efficient utilization of active sites. After functionalization, the MOF can be further grafted with active functional groups such as amines and carboxylic acids, providing feasible pathways for the covalent conjugation or non-covalent anchoring of bio-recognition elements such as nucleic acid probes and antibodies. This design strategy significantly enhances the loading capacity and luminescence quantum efficiency of ECL emitters, thereby optimizing the sensitivity and stability of the ECL sensing system and establishing MOFs as an ideal carrier matrix for constructing ECL emitters. By precisely tuning the pore size and chemical microenvironment of MOFs, efficient loading and uniform dispersion of ECL-active species can be achieved, effectively suppressing the aggregation of active materials and further improving the electron-transport efficiency and signal stability of the sensor. This provides a solid structural foundation and material support for the development of highly sensitive and interference-resistant ECL detection systems.

Relevant studies have demonstrated the significant advantages of this design strategy. Research on the design and application of lanthanide-based MOFs modified with polycyclic aromatic hydrocarbon derivatives (PTCA-La-MOFs) has shown that their nanostructure enables efficient loading of PTCA, effectively overcoming its inherent drawbacks of poor aqueous stability and tendency to aggregate. Additionally, the increased spacing between ligand molecules mitigates the aggregation-induced quenching effect, resulting in a 2.4-fold enhancement in ECL efficiency compared to PTCA aggregates. Based on this, an ECL immunoassay sensor was constructed using NG-PEI-NiZn MOFs as the sensing substrate and PTCA-La-MOFs as the signal amplification component. Leveraging the large specific surface area and excellent metallic conductivity of NG-PEI-NiZn MOFs, combined with the signal amplification capability of PTCA-La-MOFs, this sensor achieved highly sensitive and selective detection of ZEN [83].

**Figure 4:**
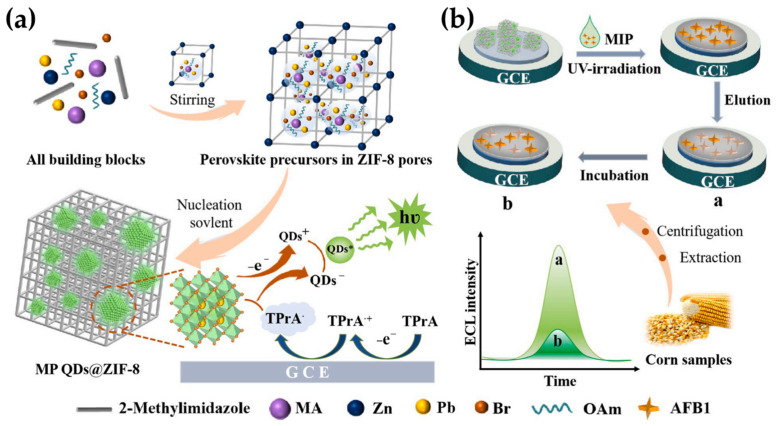
Schematic of design strategies for MOFs as carriers: (**a**) Synthesis steps and the possible ECL reaction mechanism of MP QDs@ZIF-8 nanocomposites; (**b**) Signal responses of the AFB1-imprinted ECL sensor during the whole detection procedure (reproduced with permission from Ref. [84]. Copyright 2023, Elsevier). Abbreviations: MIP, molecularly imprinted polymer; OAm, oleylamine. The symbol * represents the excited state of quantum dots (QDs) in the original literature.

Research utilizing ZIF-8 as an ECL carrier further expands the application scope of MOFs. The MP QDs@ZIF-8 composite was prepared by encapsulating methylammonium lead perovskite quantum dots (MP QDs) within the ZIF-8 framework via a pore-confinement strategy, and an AFB1 sensor was constructed by integrating molecular imprinting technology (Figure 4) [84]. The construction of Fe-MIL-101-based composite systems highlights the potential of MOFs in synergistically enhancing ECL signals. In this strategy, the luminescent reagent *N*-(4-Aminobutyl)-*N*-ethylisoluminol (ABEI) is loaded onto the MOF through amide bonds during synthesis, followed by in situ reduction to generate AuNPs, forming a Fe-MIL-101@ABEI@AuNPs composite. A thiol-modified aptamer is then immobilized on the material surface via Au-S bonds as the recognition element. This composite leverages the high porosity and large specific surface area of Fe-MIL-101 to increase ABEI loading, while the excellent conductivity of AuNPs synergistically amplifies the ECL signal [85]. In the application of Ru-based MOFs, an isomolar linear ligand exchange strategy has been employed to achieve the room-temperature green synthesis of mesoporous Zr-based MOFs. The resulting mesoporous cage structure of these Zr-based MOFs enables the single-molecule-level in situ encapsulation of Ru(bpy)_3_^2+^. The window structure of the cages prevents the leakage of Ru(bpy)_3_^2+^, while the pore enrichment effect significantly enhances the ECL efficiency. Furthermore, the covalent immobilization of polyethyleneimine (PEI) on the MOF’s surface further optimizes electron transport properties, achieving ECL self-enhancement [86]. Additionally, the construction of Ru(bpy)_3_^2+^-functionalized Ru-MOFs and their application in electrodeposition-modified electrodes not only overcomes the technical limitation of poor aqueous stability associated with traditional Ru^2+^ complexes but also provides an excellent carrier platform for hairpin DNA H1, offering new avenues for the diversified design of ECL sensing systems [87].

### 3.2. MOFs as Emitters

When MOFs serve as ECL emitters, their luminescence arises from the synergistic contribution of the metal ions/clusters and the organic linkers. Commonly used luminescent metals include Ru, Zn, Co, and Ln elements. Among these, self-emissive Ln MOFs represent a highly promising class of intrinsic ECL materials, with their luminescent properties significantly optimized through precise structural design.

**Figure 5:**
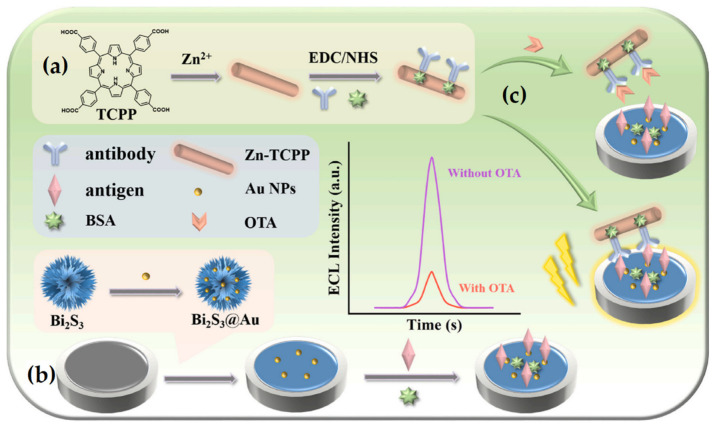
Schematic of design strategies for MOFs as emitters. (**a**) Preparation of Zn-TCPP/Ab immunosensor probe; (**b**) Assembly of the immunosensor substrate; (**c**) Mechanism of competitive immunodetection. Reproduced with permission from Ref. [53]. Copyright 2024, Elsevier.

In practical sensing applications, a Bi_2_S_3_@Au nanoflower-modified electrode serves as the substrate, enabling the immobilization of OTA antigen via Au-N bonds. Zn-TCPP is conjugated with an OTA antibody to form a signaling probe. In the absence of OTA, the probe specifically binds to the antigen on the electrode surface, generating a strong ECL signal. When OTA is present in the system, it competes with the immobilized antigen for antibody binding, resulting in a reduced number of probes bound to the electrode surface (Figure 5). Consequently, the ECL signal decreases in a regular manner with increasing OTA concentration, allowing for the quantitative detection of OTA [53]. In this system, Zn-TCPP improves the ECL response by reducing the aggregation of free porphyrin, while Bi_2_S_3_@Au nanoflowers provide a conductive and high-surface-area interface for antibody/antigen immobilization. Therefore, the main improvement is reflected in signal stability and analytical sensitivity, with a linear range of 0.0004–500 ng/mL and a LOD of 0.13 pg/mL for OTA. Significant progress has also been made in intrinsic ECL-emissive MOFs based on luminescent ligands. Using a pyrene-based polycyclic aromatic hydrocarbon derivative, 1,3,6,8-tetra(4-carboxybenzene)pyrene (H_4_TBAPy), as the luminescent ligand, a rigid MOF is self-assembled through coordination bonds with Dy^3+^. The luminescent units are inherent components of the material itself, eliminating the need for additional loading of exogenous luminescent molecules. These MOFs can directly function as ECL signal emitters for ECL [45]. This design simplifies the preparation of ECL emitters and helps avoid uneven distribution or leakage of externally loaded luminophores. However, its sensing performance still depends on charge transfer and co-reactant diffusion within the MOF. The synthetic strategies for novel intrinsic ECL emitters continue to evolve. A new intrinsic ECL emitter, NiRu MOFs, can be synthesized via a two-step hydrothermal method. First, constructing a layered Ni-MOF based on terephthalic acid coordination, followed by ion exchange to reassemble ruthenium-pyridine complexes within the framework, achieving atomic-level dispersion of ruthenium. Compared to pure Ni-MOFs, this approach significantly enhances ECL emission efficiency while simultaneously offering high and stable ECL transduction performance [88]. The improvement mainly comes from the atomically dispersed Ru sites and the interaction between Ni and Ru, which promotes ECL transmission efficiency.

The design of low-potential ECL emitters offers a novel pathway for developing biocompatible sensing systems. A Tb-BTB lanthanide-based MOF material can be synthesized via a mild hydrothermal method using 1,3,5-tris(4-carboxyphenyl)benzene (H_3_BTB) as the organic ligand and Tb^3+^ as the metal node. Leveraging the energy transfer mechanism between the ligand and the lanthanide node, this MOF achieves low-potential anodic ECL emission in a system with triethanolamine (TEOA) as the co-reactant. The onset potential is as low as +0.55 V, with a peak potential at +0.75 V, effectively mitigating the damage to biomolecular activity and reducing background interference associated with high applied potentials [89]. In addition, an intrinsic ECL emitter based on a pyrene-based MOF (PMOF) can be constructed using H_4_TBAPy as the ligand and Mg^2+^ as the metal node. The rigid framework enables aggregation-induced ECL (AI-ECL) enhancement, effectively suppressing aggregation-caused quenching and offering good acid-base tolerance. This material exhibits excellent luminescent performance in a K_2_S_2_O_8_/H_2_O_2_ dual co-reactant system, and the ECL signal can be further amplified by incorporating FeNi alloy (FNA) to accelerate radical generation. An immunoassay sensor developed based on this material enables highly sensitive target detection, providing a new strategy for the design of AI-ECL-type MOFs emitters and their application in sensitive sensing [39].

Overall, these studies show that MOF-based ECL emitters improve sensing performance through different routes, including signal stabilization, low-potential emission, improved selectivity, simplified operation, and co-reactant-assisted signal amplification.

### 3.3. MOFs as Electrocatalysts for Co-Reactant Activation

The introduction of co-reactant accelerators represents a significant breakthrough in the field of ECL. The core feature is the expansion of the ECL system from a traditional binary system composed of a luminophore and a co-reactant to a multi-component system where the emitter, co-reactant, and accelerator work synergistically. These accelerators enhance the accuracy and reliability of detection by modulating the reaction kinetics, while also deepening the theoretical understanding of the fundamental photophysical and electrochemical processes in ECL.

**Figure 6:**
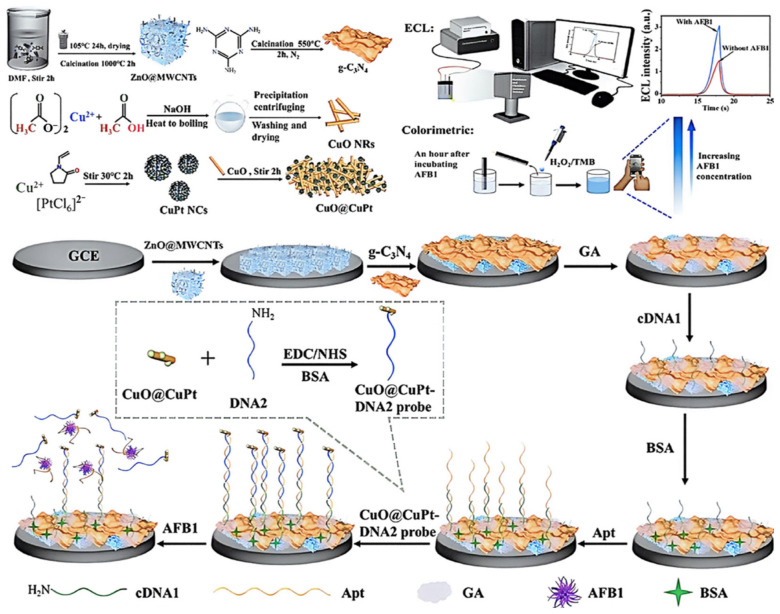
Schematic of design strategies for MOFs as electrocatalysts for co-reactant activation. Reproduced with permission from Ref. [90]. Copyright 2024, Elsevier.

MOFs have emerged as an ideal platform for constructing co-reactant accelerators, owing to their structural designability and functional versatility. The synergistic promotion is primarily achieved through two core mechanisms: catalytic enhancement and confinement effects. In terms of catalytic enhancement, MOFs can construct efficient catalytic centers that significantly accelerate the activation of co-reactants. For example, ZnO-based MOFs, when used as a co-reactant accelerator and combined with multi-walled carbon nanotubes (MWCNTs) to form a ZnO@MWCNTs heterojunction, in conjunction with graphitic carbon nitride (g-C_3_N_4_) as the emitter, enable the construction of a dual-mode ECL sensor for the detection of AFB1. The ZnO MOFs efficiently catalyze the reduction of the co-reactant persulfate, generating sulfate radicals (SO_4_·^−^) in situ, which significantly enhances the ECL signal intensity of g-C_3_N_4_ (Figure 6). By integrating subsequent CuO@CuPt probes that provide both signal quenching and colorimetric catalytic functions, this system achieves highly sensitive, multi-signal output detection of AFB1 [90]. Through molecular-level, precise design of catalytic centers, the activation efficiency of accelerators can be further optimized. In the NU-1000 MOF system, a Ru-OH-Zr synergistic catalytic center was constructed at the Zr nodes via a post-synthetic modification strategy. This center utilizes the Lewis acidity of Ru and the synergistic proton transfer effect with adjacent Brønsted acid sites to achieve efficient activation of persulfate, rapidly generating highly reactive radicals and significantly enhancing the ECL emission efficiency of the ligand [91]. Copper-doped terbium-based metal–organic frameworks (Cu: Tb-MOFs), employing isophthalic acid (IPA) as the ligand, introduce a rich Cu^2+^/Cu^+^ redox couple through Cu^2+^ doping. This not only serves as an efficient catalytic center for activating the K_2_S_2_O_8_, generating a large amount of SO_4_·^−^, but also, upon the introduction of H_2_O_2_, enables a dual-promotion effect, further increasing the concentration of reactive intermediates and significantly amplifying the ECL signal. Meanwhile, Cu: Tb-MOFs themselves can generate characteristic ECL emission through ligand-to-metal energy transfer (LMET). This synergistic combination of catalytic promotion and intrinsic luminescence greatly enhances the signal output intensity and signal-to-noise ratio, providing important insights for the design of multifunctional MOFs that integrate high catalytic activity with strong luminescent performance in co-reactant accelerator-based ECL systems [92].

### 3.4. Applications of MOFs in ECL-RET Systems

As an efficient sensing signal modulation technique, ECL-RET has been widely applied in high-sensitivity detection fields. Its core mechanism relies on spectral overlap between the donor and the acceptor. When the emission spectrum of the donor effectively overlaps with the absorption spectrum of the acceptor, energy transfer can occur, leading to either enhancement or quenching of the ECL signal, thereby enabling precise identification and quantitative analysis of the target analyte [93].

**Figure 7:**
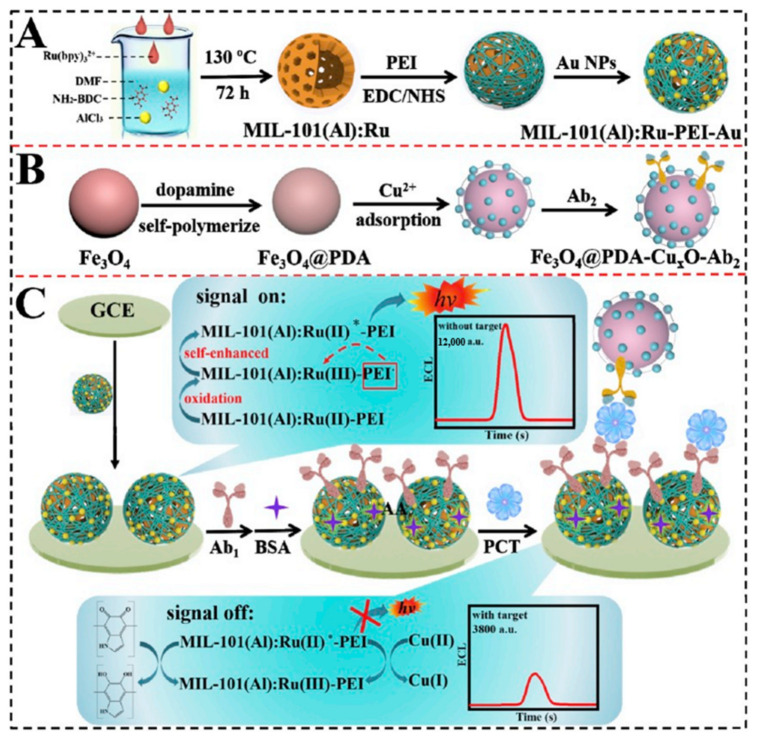
Schematic of design strategies for applications of MOFs in ECL-RET systems: (**A**) the synthesis steps of MIL-101(Al): RuPEI-Au; (**B**) the synthesis steps of Fe_3_O_4_@PDA-Cu_x_O-Ab_2_; (**C**) the fabrication process of PCT sensor and the possible luminescence mechanism. Reproduced with permission from Ref. [94]. Copyright 2024, Elsevier. The symbol * represents the excited state of the luminophore.

In mycotoxin detection, bimetallic porphyrin metal–organic frameworks (CM-MOFs) have been designed as ECL-RET quenching units, demonstrating excellent signal modulation performance. This material achieves efficient ECL signal quenching through the synergistic effect of free-radical scavenging and resonance energy transfer, significantly enhancing the detection sensitivity of ECL aptasensors (Figure 7). The sensor constructed based on this strategy exhibits an LOD of 0.37 pg/mL for ZEN and shows good accuracy and applicability in the analysis of real samples such as corn meal, providing a versatile ECL sensing strategy for the rapid and accurate detection of similar mycotoxins in food matrices [94]. The spectral matching between the ECL-RET donor and acceptor is crucial for achieving efficient energy transfer. In the case of PCN-222 MOFs constructed by coordinating Zr^4+^ with TCPP, the ECL emission spectrum shows a high degree of overlap with the absorption spectrum of the AuNPs/NSG acceptor, effectively triggering the energy transfer process. In the construction of a competitive immunosensor for ZEN, in the absence of the target, the acceptor is in close proximity to the donor surface, inducing ECL signal quenching. When ZEN is present, it competes with the antigen for antibody binding, increasing the distance between the acceptor and donor, thereby reducing the ECL-RET efficiency and restoring the signal. This sensor exhibits a wide linear response range (0.0005–1000 ng/mL) and an extremely low LOD (0.15 pg/mL), enabling precise detection of ZEN in cereal matrices such as wheat and corn [54].

Although the main focus of this review is mycotoxin detection in food, representative studies involving other analytes are also discussed in this section to highlight the general design strategies and broader applicability of MOF-based ECL-RET systems. Ru@Zn oxalate MOFs, prepared by encapsulating Ru(bpy)_3_^2+^ within zinc oxalate MOFs, and AuCoFe MOFs modified with gold nanoparticles serve as the ECL-RET energy donor and acceptor, respectively. By precisely controlling the spectral overlap between the two MOFs, efficient resonance energy transfer can be achieved, offering a novel combination of MOF-based functional materials for ECL-RET systems and enriching the material library of this sensing platform [95]. The introduction of AIECL materials has provided new insights into the design of ECL-RET donors. TPE-UiO-66, constructed by incorporating the AIE molecule tetraphenylethylene (TPE) into the UiO-66 framework, combines excellent AIECL performance with the structural characteristics of MOFs. As an ECL-RET energy donor, this material offers a stable and high-intensity ECL signal foundation. Through the precise modulation of a DNA Y-shaped structure, adjustable distances can be achieved between the donor and various energy acceptors such as gold nanoparticles and doxorubicin, enabling multiple switchable ECL-RET processes and facilitating specific target detection with controllable signal output [96]. The integration of clustered regularly interspaced short palindromic repeats (CRISPR)/Cas systems with ECL-RET technology has further enhanced the sensitivity and specificity of sensing platforms. Beyond serving as a recognition element, Cas12a offers sequence-specific cleavage activity that can amplify the analytical response, which helps improve selectivity and achieve lower detection limits. In one reported platform, rare-earth yttrium (Y-BTC) MOFs acted as the ECL-RET donor and AuNPs as the acceptor. After pre-reduction, the ECL emission of Y-BTC was strengthened. Once the target was present, Cas12a was activated and cleaved the DNA attached to the AuNPs, generating bare AuNPs that moved close to the donor surface through electrostatic interaction and produced effective RET quenching. This arrangement increased the sensitivity of the assay and, at the same time, avoided complicated electrode modification, making the sensing process more straightforward [97].

### 3.5. Multifunctional MOFs Platforms for Signal Amplification and Multiplex Sensing

**Figure 8:**
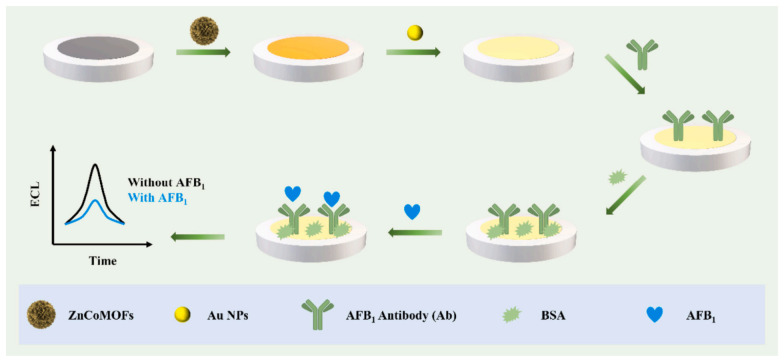
Schematic of design strategies for multifunctional MOF platforms for signal amplification and multiplex sensing. Reproduced with permission from Ref. [98]. Copyright 2025, Elsevier.

With the development of ECL sensors toward higher sensitivity and more diversified analytical functions, MOFs serving only a single role are often insufficient to satisfy the requirements of advanced sensing platforms. In this section, multifunctional MOFs refer specifically to MOF-based systems that can simultaneously play two or more roles in ECL sensing, such as serving as an ECL emitter carrier, an intrinsic ECL emitter, a co-reactant promoter, and/or an ECL-RET energy-transfer platform. The multifunctionality of MOFs arises from their tunable framework structures, high porosity, accessible active sites, and versatile host-guest integration capability. These features allow MOFs to load luminophores within their pores, generate ECL emission through luminescent ligands or metal centers, accelerate co-reactant activation via catalytic sites, and organize donor-acceptor pairs for efficient energy transfer. Among these systems, MOF composites with highly reactive surfaces for biomarker detection have shown significant progress. Using the luminescent ligand 2,3,6,7,10,11-hexahydroxytriphenylene (HHTP) as the organic linker and zinc and cobalt as metal nodes, self-amplifying conductive MOFs were synthesized via a one-step solvothermal method. In this platform, the conjugated HHTP ligand enables the MOF to act as an ECL emitter, while cobalt ions facilitate the co-reactant catalytic cycle, allowing the material to simultaneously function as a co-reactant promoter. The enhanced conductivity further improves charge transfer and ECL emission efficiency. Based on this dual-functional platform, a label-free immunosensor was developed for AFB1 detection, achieving a low detection limit of 0.31 pg/mL together with good stability and reliability in real sample analysis [98]. The design and construction of amino-functionalized MOF-based multifunctional integrated materials provide a novel strategy for developing high-efficiency MOF-based ECL emitters. Using a glutamic acid-Cr^3+^ coordinated MOF as the matrix, the in situ anchoring of Pd nanoclusters and covalent conjugation with RuP integrates several functions into one platform. Here, the MOF serves as an ECL emitter carrier for RuP, while Pd nanoclusters promote interfacial photoelectron transfer, and the whole hybrid architecture enhances ion-annihilation efficiency (Figure 8). As a result, this system achieves a 214-fold self-enhancement of the ECL signal [99]. Zr MOFs@PEI@AuAg NCs composite is a typical application of the multifunctional integration strategy in MOFs. In this system, the Zr MOFs simultaneously integrate three core functionalities: serving as an ECL emitter with H_4_TCBPE as the ligand (emission wavelength at 535 nm), providing a co-reactant facilitation effect through surface-modified PEI, and efficiently loading AuAg NCs (emission wavelength at 644 nm) within its porous structure while optimizing electron transport efficiency [100].

## 4. ECL Sensing Mechanisms Mediated by MOFs

Based on the core functional roles of MOFs in ECL sensors, including serving as a carrier, luminescent emitter, and electrocatalyst, the ECL signal generation process involving MOFs can be categorized into four fundamental mechanisms: anodic oxidation, cathodic reduction, energy transfer, and electrocatalysis. The anodic/cathodic mechanisms define the basic reaction patterns of MOF-based ECL systems, the energy transfer mechanism determines the energy flow within the system, and the electrocatalytic mechanism provides the kinetic support for the relevant reaction processes. These four mechanisms work synergistically to enable the efficient construction and performance optimization of MOF-based ECL systems.

### 4.1. Anodic ECL Mechanism Based on MOF Systems

Anodic ECL based on MOF systems refers to the process in which the MOF itself, the luminescent emitter or co-reactant loaded onto the MOF, undergoes oxidation under anodic potential to generate radical ions or other reactive intermediates. These intermediates subsequently form excited states through electron and/or energy transfer, followed by radiative transitions that emit light [29,101]. Compared to conventional anodic ECL systems, MOFs can not only serve as emitters or emitter carriers but also leverage their high specific surface area, tunable pore structures, and designable metal node/ligand architectures to facilitate reactant enrichment, interfacial charge transfer, and co-reactant catalytic oxidation, thereby significantly enhancing the ECL signal intensity and stability [102,103,104].

Currently, the anodic ECL mechanisms involving MOFs can be broadly categorized into several types. The first type is anodic ECL in which the MOF itself participates in light emission. Certain MOFs contain luminescent ligands or luminescent metal centers such as Eu^3+^ and Tb^3+^, and can generate ECL signals under anodic potential through ligand-centered or metal-centered oxidation-electron transfer processes [101,105]. However, such systems often exhibit limited luminescence efficiency and typically require enhancement strategies such as co-reactants, defect engineering, or energy transfer mechanisms [51,106,107]. The second type is indirect anodic ECL, where luminescent emitters are loaded onto MOFs. MOFs can incorporate emitters such as Ru(bpy)_3_^2+^, AIE molecules, or quantum dots through pore encapsulation, electrostatic adsorption, coordination fixation, or covalent bonding. Their high specific surface area and tunable pore structure enable efficient emitter immobilization, reactant enrichment, and quenching suppression [60,108,109]. Taking the classic Ru(bpy)_3_^2+^ and tri-*n*-propylamine (TPrA) ECL system as an example, anodic oxidation first generates Ru(bpy)_3_^3+^ and TPrA radical intermediates. Subsequently, a homogeneous electron transfer process regenerates Ru(bpy)_3_^2+^, which emits photons upon returning to its ground state [110]. The third category is enhanced anodic ECL through MOF-catalyzed oxidation of co-reactants. MOFs containing active sites such as Cu or Zn can facilitate the electrochemical oxidation of co-reactants, generating a greater number of radical intermediates capable of participating in subsequent electron-transfer processes. This leads to an increased yield of excited-state species and amplification of the ECL signal [111,112]. Furthermore, by introducing conductive ligands, constructing defect sites, or forming composites with conductive materials, the charge transport and catalytic efficiency can be further improved [106,113,114]. The fourth category is analyte-mediated anodic ECL mechanisms. In sensing systems, the target analyte can participate in the ECL process as a co-reactant, electron donor, or quencher. Alternatively, it may regulate the luminescence intensity by competing for oxidation, consuming radicals, disrupting the MOF structure, or affecting interfacial charge transfer. By leveraging the pore confinement and ligand-specific recognition capabilities of MOFs, selective detection of mycotoxins can be achieved [44,115,116]. Overall, the core of MOF-based anodic ECL is not a simple “oxidation-to-emission” process, but rather a coupled cascade involving electrode oxidation, radical generation, electron/energy transfer, and radiative transition of the excited state.

### 4.2. Cathodic ECL Mechanisms Based on MOF Systems

As a complementary pathway to anodic ECL systems, cathodic ECL has attracted continuous attention in sensing applications in recent years, primarily due to its advantages, such as lower driving potentials, better biocompatibility, and relatively easier control of the radical reaction processes [117]. Cathodic ECL typically initiates with electron injection from the electrode to the luminophore or co-reactant under negative potential conditions, triggering the reduction in relevant components and generating active intermediates. For example, S_2_O_8_^2−^ can be reduced to SO_4_·^−^ at the cathode, which subsequently promotes the formation of an excited state in the luminescent system through electron transfer or energy transfer processes, ultimately resulting in radiative emission [118]. Unlike anodic ECL, which is initiated by oxidation reactions, cathodic ECL is dominated by reduction processes; therefore, the two mechanisms exhibit strong complementarity in terms of applicable potential ranges, types of key intermediates, and compatible luminescent systems [119].

In cathodic ECL systems, MOFs can simultaneously serve multiple roles, including as luminophores, carriers, and electrocatalytic platforms. As luminophores, the metal nodes or organic linkers within MOFs can directly participate in the electrochemical excitation process, achieving luminescence through ligand-to-metal energy transfer [60]. As carriers, MOFs, with their high specific surface area and ordered pore structures, can effectively load luminescent units such as quantum dots. Additionally, the pore confinement effect enables the enrichment of co-reactants or target molecules, thereby enhancing the luminescence efficiency and signal response of the system [120]. As electrocatalytic materials, the active metal sites (e.g., Cu, Co) in MOFs can promote the reduction and activation of co-reactants such as persulfate under cathodic conditions, accelerating the generation of radical intermediates and further amplifying the ECL signal [121,122].

In addition, MOFs possess tunable structural compositions, abundant multiple active sites, and well-ordered pore arrangements, which are beneficial for improving interfacial mass transfer processes, enhancing molecular recognition efficiency, and increasing the reproducibility of detection results. However, MOF materials generally suffer from inherently poor electrical conductivity, and in practical applications, it is often necessary to improve this property through strategies such as compositing with conductive substrates or incorporating conductive linkers [123,124]. Currently, cathodic ECL systems based on MOFs have demonstrated promising application potential in various fields, including bioanalysis, environmental monitoring, and food safety detection, particularly showing excellent prospects for the highly sensitive detection of mycotoxins such as AFB1 and OTA [125,126].

### 4.3. Energy Transfer Mechanisms in MOF-Mediated ECL Systems

MOFs play three principal roles in ECL energy transfer systems: carriers, connectors, and modulators [127,128,129]. Through strategies such as pore confinement, energy-level alignment, and interface regulation, MOFs can reduce energy-transfer barriers within the system, facilitating directional energy transfer between emitters, between emitters and co-reactant intermediates, and between emitters and quenchers. This effectively modulates the efficiency of excited-state generation and influences the intensity, stability, and emission behavior of the ECL signal [130,131,132,133]. The key lies in leveraging the framework structure to construct efficient donor-acceptor transfer pathways. Current research indicates that MOF-mediated energy transfer processes mainly include ECL-RET, Electron transfer-coupled energy migration (ETEM), and Förster resonance energy transfer (FRET), among others [133,134,135].

Among these, ECL-RET primarily relies on the ordered pores of MOFs to confine the donor-acceptor distance and optimize spectral overlap, thereby enhancing non-radiative energy transfer efficiency [93]. ETEM is closely associated with the catalytic activity of metal nodes, coupling electron transfer with energy migration while promoting the generation of reactive intermediates from co-reactants [91]. FRET imposes stricter requirements on donor-acceptor distance and spectral matching, for which the tunable pore size and rigid framework of MOFs provide an ideal structural basis [136,137]. Therefore, the advantages of MOF-mediated energy transfer are mainly reflected in controllable spatial separation between donors and acceptors, facile optimization of energy level alignment, high framework stability, and strong multifunctional integration capability.

### 4.4. Electrocatalytic Mechanisms in MOF-Mediated ECL Processes

Electrocatalytic mechanisms refer to the process in which MOFs, leveraging their abundant metal active sites, well-defined pore confinement effects, and tunable energy level structures, function as electrocatalysts or catalytic platforms to precisely regulate ECL systems. This regulation influences the reaction rates and pathways of key steps, such as co-reactant activation, electron transfer, and excited-state generation, ultimately leading to enhanced ECL signal intensity, improved stability, or optimized selectivity [90].

Studies indicate that MOFs may promote electrocatalysis in ECL processes through two primary aspects: first, metal nodes or functionalized ligands can act as catalytically active centers, participating in the redox activation of co-reactants and facilitating the generation of intermediates such as radicals, thereby creating favorable conditions for subsequent excited-state formation [138]. Second, MOFs possess high specific surface areas and well-ordered pores, which enable the enrichment of luminophores, co-reactants, and target molecules, shorten mass transfer distances, reduce intermediate loss, and enhance the probability of interfacial reactions [139]. MOF-mediated electrocatalysis mainly operates in the early stage of ECL reactions, particularly during the activation of co-reactants and the supply of reactive intermediates. For instance, in systems where persulfate or H_2_O_2_ serves as a co-reactant, MOFs can promote their electrochemical transformation, thereby improving the efficiency of radical generation and excited-state formation [140,141]. Consequently, the electrocatalytic mechanism primarily regulates the reaction kinetics, while the energy-transfer mechanism determines the efficient delivery of excitation energy; the two mechanisms collectively influence the output performance of ECL sensors.

## 5. Applications of MOF-Based ECL Sensors for Mycotoxin Detection in Food

A wide variety of mycotoxins exist in food, among which AFB1, OTA, ZEN, DON, and FB1 are common and representative examples. Mycotoxins are secondary metabolites produced by fungi growing in food or feed, primarily contaminating agricultural products such as corn, wheat, and peanuts [142]. Approximately 25% of the world’s grain is contaminated with mycotoxins annually, and the Food and Agriculture Organization of the United Nations estimates that the resulting economic losses amount to hundreds of billions of US dollars each year [143]. The high sensitivity and low background noise characteristics of ECL technology, combined with the functionalization potential of MOFs, enable the construction of detection platforms that integrate high selectivity, high sensitivity, and portability, offering a novel solution for the accurate and rapid detection of mycotoxins.

### 5.1. AFB1

*Aspergillus flavus* (AFs) is widely distributed in soil, grains, and feed, and it readily proliferates under warm and humid conditions, particularly in crops such as corn and peanuts [144,145]. Among the toxins it produces, AFB1 is the most toxic and is classified as a Group 1 carcinogen, exhibiting carcinogenic, mutagenic, and teratogenic effects [146]. Because of its high toxicity, chemical stability, and frequent occurrence in food commodities, AFB1 remains one of the most critical mycotoxins requiring strict monitoring [147].

For AFB1 detection, the reported MOF-based ECL sensing strategies are mainly designed around aptamer- or antibody-mediated recognition, including competitive assays, target-triggered DNA displacement/amplification strategies, and signal-on/off formats. This design choice is largely related to the small-molecule nature of AFB1, which makes it less suitable for conventional sandwich-type assays. In these platforms, MOFs are selected not only for their porous structures, but also for their multifunctional roles in probe immobilization, luminophore loading, electron-transfer regulation, and signal amplification. This design enables the combination of molecular specificity and ultrasensitive ECL readout, which is particularly important for trace-level AFB1 analysis. In addition, some studies have introduced ratiometric, dual-signal, or enzyme-assisted nucleic acid amplification modes to improve analytical reliability and sensitivity in complex food matrices.

As summarized in Table 1, several MOF-based ECL sensors for AFB1 have achieved very low LODs and have been successfully tested in real samples such as edible oil, peanut, and cornmeal. For example, an RI-MOF-based aptameric ECL biosensor was constructed with Nb.BbvCI-assisted DNA walker amplification on a RI-MOF/PANI NRs/rGO-modified GCE. The system achieved an LOD of 1.35 pg/mL and a linear range of 0.01–100 ng/mL for AFB1 detection. In this design, the coreactant-free annihilation ECL RI-MOF emitter improved ECL efficiency, while the target-triggered DNA walker amplification enhanced sensitivity. The sensor was successfully applied to rice flour and wheat flour samples, and the obtained results were statistically consistent with ELISA analysis [57]. These results confirm the promising application potential of MOF-based ECL platforms for rapid AFB1 screening. Nevertheless, many of the currently reported assays still involve multistep fabrication and incubation procedures, and most real-sample studies rely on spiked recovery experiments rather than systematic comparison with reference chromatographic methods. Therefore, future efforts should focus on simplifying operation, improving reproducibility and long-term stability, and promoting scalable fabrication for routine food-safety monitoring.

### 5.2. OTA

OTA is primarily produced by several toxigenic fungal species belonging to the genera *Aspergillus* and *Penicillium*, including *Aspergillus ochraceus* and *Aspergillus carbonarius* [148]. Due to its chemical stability, OTA is difficult to eliminate through conventional processing or standard storage methods, allowing it to persist throughout the food chain. Chronic exposure to low doses of OTA may further impair immune defense mechanisms, induce chronic liver damage, and increase the risk of associated cancers. Therefore, OTA is considered a significant contaminant requiring ongoing attention in the fields of food safety and public health [149].

In the case of OTA, recent MOF-based ECL sensors appear to place greater emphasis on signal reliability in complex matrices rather than sensitivity alone. OTA is frequently monitored in coffee, wine, cereals, and dried fruits, where pigments, polyphenols, proteins, and other coexisting substances may interfere with ECL emission, electron transfer, or surface recognition events. To address these matrix-related challenges, reported platforms often combine OTA aptamers with MOF-enabled signal regulation, including ratiometric ECL responses, target-induced quenching/recovery, and energy- or electron-transfer modulation. Here, MOFs function as tunable interfacial architectures rather than passive porous carriers: they can stabilize ECL emitters, organize recognition probes, regulate local electron-transfer processes, and provide internal reference or amplification effects. These features make MOF-based ECL systems particularly useful for OTA detection when both trace-level sensitivity and resistance to matrix-induced signal fluctuation are required.

Table 1 shows that MOF-ECL methods for OTA are not all designed with the same objective. Some aim to push the detection limit lower, while others are more concerned with keeping the signal stable in complex food samples. Fan et al. [53] developed a competitive ECL immunoassay using Zn-TCPP MOF nanorods as cathodic luminophores and Bi_2_S_3_@Au nanoflowers as the electrode substrate. Incorporating TCPP into the Zn-based MOF helped suppress porphyrin aggregation, and the Bi_2_S_3_@Au layer improved interfacial conductivity. The resulting sensor showed a linear range of 0.0004–500 ng/mL and a detection limit of 0.13 pg/mL. It also performed well in spiked wheat flour and feed samples, with recoveries ranging from 90.1% to 109.2%. Still, the studies summarized in Table 1 suggest that most current OTA assays have mainly been tested in spiked samples. Further work is needed to improve resistance to matrix fouling, simplify sample preparation, and verify performance in naturally contaminated foods.

### 5.3. ZEN

ZEN is a typical estrogenic mycotoxin produced by Fusarium. It is mainly found in cereal crops such as corn, wheat, and rice, as well as their derived products, and may also enter animal-derived foods through contaminated feed. Owing to its stable physicochemical properties, ZEN is resistant to degradation during grain storage and food processing. After ingestion, it can be absorbed through the gastrointestinal tract, metabolized mainly in the liver and intestine, and retained for a prolonged period due to enterohepatic circulation. Its toxicity is closely associated with estrogenic effects, including reproductive dysfunction, hormonal imbalance, and developmental abnormalities, and long-term exposure may also induce hepatotoxic, immunotoxic, and genotoxic effects [150].

As summarized in Table 1, Fan et al. [54] developed a competitive ECL-RET immunosensor for ZEN detection using PCN-222 as the ECL donor and AuNPs/NSG as the acceptor. PCN-222 was selected because of its stable ECL emission, while AuNPs/NSG enabled efficient signal quenching through spectral overlap and also facilitated antibody immobilization. The sensor showed a wide linear range of 0.0005–1000 ng/mL and a low LOD of 0.15 pg/mL. It was successfully applied to wheat and corn samples, with recoveries of 86.8–104.6%, and the results agreed well with HPLC-FLD. Although this strategy improves sensitivity and simplifies signal readout compared with chromatographic methods, it still requires electrode modification and sample pretreatment. Future work should focus on simpler fabrication and validation in naturally contaminated cereal samples.

### 5.4. Detection of Other Common Mycotoxins

In addition to AFB1, OTA, and ZEN, DON and FB1 are also important Fusarium-derived mycotoxins frequently found in cereal and grain-based foods. DON mainly contaminates wheat, corn, and their derived products, and is difficult to remove during conventional processing because of its high thermal and chemical stability. Exposure to DON may cause gastrointestinal symptoms, immune dysfunction, and other systemic toxic effects, making it a key contaminant in grain and feed safety [151]. FB1 is commonly produced by Fusarium verticillioides and Fusarium proliferatum and is widely detected in corn, rice, wheat, and related products. It can disrupt intestinal barrier function, hepatic metabolism, and sphingolipid metabolism, thereby inducing immunotoxicity, hepatotoxicity, neurotoxicity, oxidative stress, and apoptosis [152].

As summarized in Table 1, MOF-based ECL platforms have recently been developed for DON and FB1 detection. Nie et al. [62] used this AIECL platform for competitive DON detection, achieving a linear range of 0.007–1000 ng/mL and an LOD of 2.3 pg/mL. The sensor showed satisfactory recoveries in wheat and corn flour samples and gave results comparable to LC-MS/MS. Despite its high sensitivity, further simplification of electrode preparation and validation with naturally contaminated samples remains necessary. For FB1, Jin et al. [153] developed a visual BPE-ECL biosensor based on MB@Zr-MOFs and DNA walker-assisted amplification. Zr-MOFs acted as carriers for methylene blue to amplify the ECL signal, and the target-triggered DNA walker enabled sensitive signal modulation. The sensor detected FB1 in the range of 5 × 10^−5^–0.5 ng/mL and showed recoveries of 99.2–110.6% in maize and peanut samples. These studies demonstrate the advantages of MOF-ECL sensing in sensitivity, sample consumption, and visual or simple signal readout. Nevertheless, further simplification of electrode fabrication, probe assembly, and real-sample pretreatment is still needed, especially for naturally contaminated samples.

### 5.5. Simultaneous Detection of Multiple Mycotoxins

In real food samples, mycotoxin contamination often exhibits a “co-occurrence” pattern, where multiple toxins are present simultaneously. Although single-toxin MOF-ECL biosensors can achieve high selectivity toward individual targets through specific aptamers or other recognition elements, single-toxin detection methods are often insufficient to meet the practical requirements of food safety monitoring. This limitation arises not from poor selectivity, but because coexisting mycotoxins can exert synergistic or additive toxicity, exceeding the hazard posed by any single toxin alone [154]. Conducting separate assays for each toxin increases analysis time, sample consumption, and operational complexity [44]. Therefore, the development of simultaneous detection technologies capable of quantifying multiple mycotoxins in a single assay holds significant practical importance and application value. MOF-based ECL sensors, with their tunable structures and functionalities, offer a promising platform for the simultaneous detection of multiple mycotoxins through the rational design of recognition elements and signal transduction systems. These sensors have attracted increasing attention in recent years. Representative studies are summarized in Table 1.

**Table 1:** Applications of MOF-based ECL sensors for mycotoxin analysis in food samples.

MOF Composites	Electrode Configuration	Assay Format	Assay Time	Target	Linear Range	LOD	Food Matrices	Ref.
AuPtNPs/Ni-Co NCs	GCE	Aptasensor	3 h	AFB1	0.001–1000 ng/mL	0.76 pg/mL	Edible oil, peanut, cornmeal	[41]
RI-MOF	GCE	Aptasensor	3 h	AFB1	0.01–100 ng/mL	3.35 pg/mL	Food matrix not specified	[57]
Zr-TCPB	GCE	Aptasensor	1 h	AFB1	0.01–100 ng/mL	0.79 pg/mL	Corn and milk	[61]
MIL-88B(Fe)-NH_2_ @Ru(bpy)_3_^2+^	GCE	Sandwich immunoassay	4 h	AFB1	1 pg/mL–100 ng/mL	209 fg/mL	Corn	[68]
MP QDs@ZIF-8	GCE	MIP-based sensor	20 min	AFB1	11.55 fg/mL–20 ng/mL	3.5 fg/mL	Corn	[84]
ZnO@MWCNTs	GCE	Aptasensor	—	AFB1	1.0 fg/mL–1.0 μg/mL	0.971 fg/mL	Oat flour	[91]
Tb-TBAPy MOF	GCE	Competitive immunoassay	2 h	AFB1	0.001–1000 ng/mL	0.33 pg/mL	Wheat flour and corn flour	[155]
UiO-66-NH_2_	CNTs/PVDF	Aptasensor	130 min	AFB1	50 fg/mL–5 ng/mL	7.8 fg/mL	Corn	[119]
Ru-MOF	Aminated ITO electrode	Aptasensor	4.5 h	AFB1	1.0 fg/mL–1.0 pg/mL	0.84 fg/mL	Corn	[156]
In-MOF	GCE	Competitive immunoassay	25 h	AFB1	0.002 ng/mL–1000 ng/mL	0.42 pg/mL	Wheat flour and corn flour	[157]
MWCNTs/Fc-MOF	GCE	Competitive immunoassay	21 h	AFB1	10 fg/mL–100 ng/mL	5.39 fg/mL	Food matrix not specified	[158]
Zn-TCPP	GCE	Competitive immunoassay	14 h	OTA	0.0004 ng/mL–500 ng/mL	0.13 pg/mL	Wheat flour and feed	[53]
PDA@ZIFs	GCE	Competitive immunoassay	17 h	OTA	10.0 fg/mL–1.0 ng/mL	4.8 fg/mL	Flour and rice	[79]
NU-1000(Zr)	GCE	Competitive immunoassay	4.5 h	OTA	0.1 pg/mL–500 ng/mL	33 fg/mL	Coffee	[159]
PCN-224-Mn	GCE	Competitive immunoassay	14 h	OTA	0.0002 ng/mL–1000 ng/mL	0.067 pg/mL	Corn and wheat	[160]
ZnCoMOF	GCE	Aptasensor	15 h	OTA	0.01 pg/mL–10 ng/mL	3.98 fg/mL	Beer	[148]
PCN-222	GCE	Competitive immunoassay	14 h	ZEN	0.0005 ng/mL–1000 ng/mL	0.15 pg/mL	Wheat and corn	[54]
PTCA-La-MOF, NiZn MOF, NG-PEI-NiZn MOF	GCE	Sandwich immunoassay	18 h	ZEN	10 fg/mL–100 ng/mL	0.21 fg/mL	Corn flour, milk, cereals	[83]
Zr-MOF	GCE	Competitive immunoassay	14 h	ZEN	0.0001 ng/mL–100 ng/mL	0.034 pg/mL	Wheat flour and pig urine	[161]
Eu-PCP	GCE	Sandwich immunoassay	4 h	ZEN	0.001 μg/kg–200 μg/kg	9.75 × 10^−5^ μg/kg	Maize	[162]
MPF	GCE	Competitive immunoassay	4.5h	ZEN	0.001 ng/mL–500 ng/mL	1.03 × 10^−4^ ng/mL	Wheat and corn	[163]
CuO/NH_2_-UiO-66	GCE	Aptasensor	25 min	ZEN	0.1 fg/mL–0.5 μg/mL	0.085 fg/mL	Corn juice	[164]
Ru-MOF	FTO/CNTs	Aptasensor	5 h	DON	0.01 μg/kg–500 μg/kg	0.009 μg/kg	Wheat and corn	[58]
Eu-MOF	GCE	Competitive immunoassay	15 h	DON	0.007 ng/mL–1000 ng/mL	2.3 pg/mL	Wheat and corn flour	[62]
TiO_2_-MOF	FTO/CNTs	Competitive immunoassay r	30 min	DON	0.1 pg/mL–20 ng/mL	0.03 pg/mL	Wheat and corn	[165]
Zn-MOF	GCE	Intramolecular enhancement,	6 h	DON	0.0001 ng/mL–100 ng/mL	0.036 pg/mL	Food matrix not specified	[166]
MB@Zr-MOF	ITO electrode	Aptasensor	6.5 h	FB1	5 × 10^−5^ ng/mL–0.5 ng/mL	—	Maize and peanut	[153]
UiO66-NH_2_	GCE	Competitive immunoassay	15 h	AFB1	0.08 ng/mL–1000.0 ng/mL	26.7 pg/mL	Wheat and corn	[167]
				OTA	0.006 ng/mL–1000.0 ng/mL	2.0 pg/mL		
AgNPs@ZIF-67	Spatially resolved ITO electrode	Aptasensor	1 h	DON	0.005 μg/kg–150 μg/kg	0.00018 μg/kg	Wheat	[168]
				AFB1	0.05 μg/kg–100 μg/kg	0.00109 μg/kg		
UiO-66-NH_2_	GCE	Aptasensor	50 min	AFB1	0.01 ng/mL–1000 ng/mL	0.0062 ng/mL	Food matrix not specified	[169]
				OTA		0.0037 ng/mL	Food matrix not specified	

## 6. Conclusions and Perspectives

### 6.1. Challenges and Solutions of MOF-Based ECL Sensors

MOF materials, owing to their high specific surface area, tunable pore structures, and abundant surface functional sites, generally exhibit excellent enrichment capability, recognition performance, and selectivity, providing a robust material foundation for the efficient detection of mycotoxins. Moreover, MOF-based ECL sensing systems have demonstrated high detection sensitivity and good selectivity. However, for practical applications involving rapid identification and trace-level detection of contaminants in complex samples, both MOF materials and MOF-based ECL sensing platforms still face several critical challenges: (1) Limited Structural Stability: Although the coordination structures formed by metal ions and organic linkers provide MOFs with high specific surface area and customizable pore architecture, they also exhibit notable performance drawbacks. MOF materials are prone to hydrolysis and framework collapse under high humidity, extreme pH, or elevated temperatures, leading to compromised structural integrity [170,171]. This defect limits their practical application in the detection of food mycotoxins, as the complex food matrix (such as high humidity, acidic/alkaline environment) is likely to cause the degradation of MOFs, thereby affecting the accuracy and reliability of mycotoxin detection results. More importantly, in practical food analysis, complex food matrices generate several major interferences for MOF-ECL biosensors. These interferences mainly include (i) structural degradation of MOFs induced by moisture, acidity/alkalinity, or salts; (ii) non-specific adsorption of proteins, lipids, and other macromolecules on the MOF or electrode surface, which can block active sites and reduce signal reproducibility; (iii) electrode fouling/passivation and ECL quenching caused by pigments, phenolics, and other electroactive components; (iv) cross-interference from coexisting contaminants, ions, or structurally similar compounds, which may lead to false responses or reduced selectivity. Such matrix effects are particularly relevant in real samples such as corn, rice, wheat, and wine. Therefore, although MOF-ECL systems show excellent analytical sensitivity under laboratory conditions, matrix interference remains a major obstacle to their practical development in food mycotoxin analysis [172,173,174]. (2) Batch-to-Batch Performance Variability in Large-Scale Production: In large-scale synthesis, continuous flow synthesis techniques remain underdeveloped, with limited precision in controlling reaction parameters, leading to inconsistent performance across production batches [175]. (3) High cost and energy consumption: MOF materials rely on high-temperature and high-pressure solvothermal conditions, involve the use of large amounts of toxic, corrosive, or flammable chemicals, require long crystal growth times, and entail high costs and energy demands for large-scale reaction equipment [176]. (4) For MOF-based ECL sensors, the limited variety of aptamers restricts the performance and application scope of aptamer-MOF ECL biosensors. In addition, most current MOF-based ECL systems are still in the laboratory stage, and their commercialization and practical application face substantial obstacles [177,178].

To address the aforementioned limitations, researchers have primarily focused on optimizing MOF-based ECL systems from the following aspects. In terms of material modification, functional groups such as amino, carboxyl, or thiol groups can be introduced onto the surface of MOFs to enhance the material’s stability in practical detection environments and provide stable binding sites for the controlled immobilization of antibodies or aptamers. Alternatively, the rigidity and stability of the MOF can be improved through ligand cross-linking strategies, such as EDTA-mediated metal node chelation [179,180]. Regarding process optimization, the adoption of continuous-flow reactors should be promoted to enable precise control over parameters such as temperature and concentration, thereby ensuring batch-to-batch consistency. Specifically, continuous-flow reactors enable the fabrication of continuous, dense, and well-crystallized MOF thin films. These reactors offer advantages such as smaller reaction volumes, faster heating and cooling rates, improved mixing efficiency, and more accurate control of reaction conditions, all of which contribute to enhanced product consistency [181]. In terms of production cost, the integration of microwave-assisted synthesis with continuous flow technology represents a feasible approach at the current stage. This method significantly reduces reaction time, lowers energy consumption and solvent usage, and improves time-space yield and batch stability. Additionally, the use of low-cost metals and ligands, as well as the substitution of aqueous media for organic solvents, can effectively reduce raw material and production costs, facilitating large-scale application [176]. Future efforts should focus on developing high-efficiency aptamers to expand the applicability of MOF-based ECL systems. Meanwhile, research should be devoted to promoting practical applications by developing portable and field-deployable devices for environmental monitoring and other real-world scenarios.

### 6.2. Future Trends

To meet the demands of practical trace-level contaminant detection and ensure the stability of detection systems, future developments should focus on material optimization and innovation, portable and intelligent equipment, as well as green and standardized production. These efforts will continue to address challenges such as interference from complex matrices, insufficient stability, weak electrical conductivity, and bottlenecks in large-scale fabrication.

#### 6.2.1. Development of Novel Multifunctional MOF Materials

The directed synthesis of MOF crystals with high humidity and thermal resistance, as well as enhanced acid-base stability, can address the issue of structural collapse caused by sensitivity to water molecules and harsh acidic or basic environments in practical applications. For instance, fluorination significantly improves the hydrophobicity of metal–organic framework materials, enhancing their moisture stability in aqueous and humid conditions [182]. Additionally, controlling the degree of interpenetration within the framework can stabilize the pore structure and prevent framework collapse at elevated temperatures, thereby improving thermal stability [183]. Furthermore, the in situ encapsulation of natural clay minerals with MOFs can enhance acid-base resistance. For example, the coordination between hydroxyl groups on the surface of Palygorskite (Pal) and Fe^3+^ strengthens the structural integrity of MIL-88A(Fe), allowing it to maintain structural stability and performance over a broad pH range of 2–12 [184]. More importantly, the design of novel multifunctional MOFs is not limited to improving environmental stability, but also extends to the integration of sensing, catalytic, and antibacterial functions. Beyond mycotoxin detection, MOF-based photoelectric sensing materials show great potential in versatile applications. For instance, porphyrinic MOFs (Zn-TCPP and PCN-224) can display both fluorescence sensing and nanozyme catalytic activities [185,186]. After modifying with Au/Pt nanoparticles and functional peptides, these composites enable sensitive fluorescence recognition toward bacteria and efficient synergistic antibacterial performance. Such a multifunctional design offers a new strategy for upgrading MOF-based sensing platforms from single detection to integrated intelligent systems.

Beyond molecular-level multifunctionality, device-level integration is also crucial for advancing MOF-based sensing systems. Three-dimensional-printed electrochemical electrodes provide rapid prototyping, mask-free fabrication, material versatility, and customizable architectures, which are beneficial for miniaturized and wearable sensing platforms. Their tailored geometries can increase the effective surface area and reduce impedance, as demonstrated by skyscraper-like electrodes for sensitive biomarker detection and FDM-printed electrochemical rings for sweat glucose monitoring [187]. These advantages could synergize with MOFs by coupling the macro/mesoscale designability of 3D printing with the molecular-level porosity, tunable active sites, and host-guest recognition of MOFs. MOFs may serve as surface modifiers, enrichment layers, or components of printable composite filaments, while conductive 3D-printed frameworks provide customizable support for electron transfer. This integration may facilitate the development of portable, wearable, and all-in-one MOF-based sensing platforms.

#### 6.2.2. Development of Intelligent and Portable Sensing Platforms

Smartphones, equipped with advanced computational power, built-in sensors, wireless connectivity, data management systems, and user-friendly interfaces, have been widely adopted as integrated detectors, display units, and data processing terminals due to their high penetration rate. Research has demonstrated that a potential modulation strategy based on zinc-cobalt-nitrogen-doped carbon (ZnCoN-C) achieves zero background signal output, ensuring no stray light interference during imaging while enabling more accurate visual quantitative signal readout. The extracted RGB intensity from wide linear range and high sensitivity imaging shows a good linear correlation with the logarithm of OTA concentration, with a detection limit as low as 12.3 fg/mL, meeting the requirements for ultra-sensitive detection [148]. Additionally, Huang et al. developed a portable dual-mode paper chip based on aptamer-gated UiO-66-NH_2_ MOFs, integrating ECL and colorimetric detection for ultra-sensitive and rapid AFB1 detection. This portable detection system is compact and lightweight, eliminating the need for large instruments such as electrochemical workstations or fluorescence spectrometers, thereby enabling on-site, in situ, and point-of-care testing. Moreover, the detection process does not require expensive reagents or complex modifications, significantly reducing the cost of field testing [115]. Recent studies on paper-based ECL platforms have further enriched the design of portable biosensors. For instance, Zhu et al. [188] developed a hand-drawn paper-based bipolar electrode ECL platform using pencil-drawn graphite traces, in which the bipolar configuration spatially separated the sensing and ECL reporting units, thereby reducing signal interference while lowering fabrication cost and improving portability. More recently, paper substrates have also been exploited as functional materials for reagent preloading and storage. A recent study demonstrated that office paper can outperform highly porous filter paper in ECL sensing and can be used to store dried tripropylamine coreactant, eliminating manual reagent addition and improving operational simplicity and storage stability [189]. In summary, smartphone-controlled portable ECL detection devices, together with emerging paper-based ECL platforms featuring simplified fabrication, low-cost materials, and reagent prestorage capability, can achieve rapid visual qualitative analysis and convenient quantitative readout without the need for complex instrumentation or professional data processing.

The introduction of artificial intelligence algorithms has significantly enhanced the efficiency and accuracy of data processing and analysis. In recent years, machine learning (ML) methods have gradually demonstrated their potential in material design and sensor development, particularly beginning to be applied in the optimization of MOF-based ECL sensors. ML can analyze the relationship between structural parameters of MOFs, such as metal node types, pore size, specific surface area, and functional groups, and sensing performance metrics (e.g., sensitivity, detection limit, response time), thereby assisting in the screening of optimal material combinations and reducing the trial-and-error costs associated with traditional experimental approaches [190]. At the same time, ML can provide a robust, interference-resistant, and adaptive data analysis framework for ECL sensors, effectively addressing challenges such as signal nonlinearity, inter-electrode variability, and environmental fluctuations. Through multimodal data fusion, detection accuracy can be improved by approximately 80%, and it enables direct modeling from raw time-series data without the need for complex feature extraction, significantly enhancing the accuracy and practicality of low-cost portable ECL systems [191].

#### 6.2.3. Advancement of Green Synthesis Strategies

The traditional synthesis of MOF materials relies heavily on toxic organic solvents and corrosive additives, which can lead to environmental pollution and health hazards. Moreover, complex post-treatment processes and high costs hinder the large-scale production and practical application of MOF materials. Therefore, the development of low-toxicity, low-energy, and facile yet efficient green synthesis strategies is essential for achieving environmentally friendly, safe, cost-effective, and scalable production of MOFs, as well as for expanding their application in mycotoxin detection. The core of advancing green synthesis lies in replacing toxic reagents at the source, reducing reaction conditions, simplifying post-treatment, and enhancing biocompatibility. For example, using non-toxic reagents instead of toxic organic solvents can significantly reduce environmental impact during MOF synthesis. Employing low-temperature and short-duration reactions can dramatically decrease energy consumption compared to conventional high-temperature hydrothermal methods, enabling mild and energy-efficient preparation of MOF materials. Post-synthesis purification can be completed through simple water washing, centrifugation, and ambient drying, eliminating the need for complex steps such as solvent exchange or high-temperature activation, resulting in a more straightforward and eco-friendly process. This green synthesis strategy yields MOF materials with excellent water stability, thermal stability, and efficient antibacterial properties [192,193]. In summary, establishing a green, low-toxic, low-energy, and process-simplified MOF synthesis system not only enhances the environmental friendliness and safety of the material preparation process but also promotes the large-scale production and industrial application of MOFs in practical fields such as mycotoxin detection.

## 7. Conclusions

MOFs have emerged as an ideal platform for constructing high-performance mycotoxin sensors, leveraging their highly ordered porous structure, large specific surface area, tunable pore size, and modifiable surface properties. Studies have demonstrated that MOFs can serve as emitters, carriers, and co-reaction accelerators and play a pivotal role in ECL sensing. Their sensing strategies offer exceptional sensitivity and selectivity, with continuous optimization of detection limits and specificity. However, the translation of MOF-based ECL sensors from the laboratory to practical applications is still confronted with several challenges. MOF materials are prone to hydrolysis and structural collapse under high humidity or extreme conditions, which can compromise their integrity. Additionally, batch-to-batch performance variability is common in scale-up production, and the high synthesis cost of many high-performance MOFs poses a significant barrier to large-scale manufacturing, making the development of green and low-cost synthetic technologies critical. Moreover, while most existing MOF-based ECL sensors target individual mycotoxins, the complexity of real-world samples, where multiple toxins often coexist, highlights the need for the development of multi-analyte detection systems as a future focus. Looking ahead, the advancement of MOF-based ECL sensors toward intelligent integration is an inevitable path. On the material level, the development of functional materials with high stability, strong recognition capabilities, and reasonable cost is essential. At the device level, integration with microfluidics, paper-based, or wearable technologies will enable on-site rapid detection. Furthermore, the fusion of artificial intelligence and big data to construct an intelligent system that integrates detection, analysis, and early warning will transform the approach from “passive response” to “proactive prevention and control.” Providing a new generation of technical support for food safety and facilitating practical applications.

## Data Availability

No new data were created or analyzed in this study. Data sharing is not applicable to this article.
